# Fine mapping and epistatic interactions of the vernalization gene *VRN*-*D4* in hexaploid wheat

**DOI:** 10.1007/s00438-013-0788-y

**Published:** 2013-11-09

**Authors:** Nestor Kippes, Jie Zhu, Andrew Chen, Leonardo Vanzetti, Adam Lukaszewski, Hidetaka Nishida, Kenji Kato, Jan Dvorak, Jorge Dubcovsky

**Affiliations:** 1 Department of Plant Sciences, University of California, Davis, CA 95616 USA; 2 USDA-ARS Wheat Genetics, Quality, Physiology and Disease Research Unit, Washington State University, Pullman, WA 99164-6420 USA; 3 Grupo de Biotecnología y Rec. Genéticos, INTA EEA Marcos Juárez, Ruta 12 S/N, (2580), Marcos Juárez, Córdoba Argentina; 4 Consejo Nacional de Investigaciones Científicas y Técnicas (CONICET), Buenos Aires, Argentina; 5 Department of Botany and Plant Sciences, University of California, Riverside, CA 92521 USA; 6 Graduate School of Environmental and Life Science, Okayama University, 1-1-1, Tsushima-Naka, Kita-Ku, Okayama, 700-8530 Japan; 7 Howard Hughes Medical Institute and Gordon and Betty Moore Foundation Investigator, Chevy Chase, USA

**Keywords:** Wheat, Vernalization, *VRN-D4*, *VRN1*, Genetic mapping, *VIL*, Flowering

## Abstract

Wheat vernalization requirement is mainly controlled by the *VRN1*, *VRN2*, *VRN3*, and *VRN4* genes. The first three have been cloned and have homoeologs in all three genomes. *VRN4* has been found only in the D genome (*VRN*-*D4*) and has not been cloned. We constructed a high-density genetic map of the *VRN*-*D4* region and mapped *VRN*-*D4* within a 0.09 cM interval in the centromeric region of chromosome 5D. Using telocentric 5D chromosomes generated from the *VRN*-*D4* donor Triple Dirk F, we determined that *VRN*-*D4* is located on the short arm. The *VRN*-*D4* candidate region is colinear with a 2.24 Mb region on *Brachypodium distachyon* chromosome 4, which includes 127 predicted genes. Ten of these genes have predicted roles in development but we detected no functional polymorphisms associated to *VRN*-*D4*. Two recombination events separated *VRN*-*D4* from *TaVIL*-*D1*, the wheat homolog of *Arabidopsis* vernalization gene *VIL1*, confirming that this gene is not a candidate for *VRN*-*D4*. We detected significant interactions between *VRN*-*D4* and other four genes controlling vernalization requirement (*Vrn*-*A1*, *Vrn*-*B1*, *Vrn*-*D1*, and *Vrn*-*B3*), which confirmed that *VRN*-*D4* is part of the vernalization pathway and that it is either upstream or is part of the regulatory feedback loop involving *VRN1*, *VRN2* and *VRN3* genes. The precise mapping of *VRN*-*D4* and the characterization of its interactions with other vernalization genes provide valuable information for the utilization of *VRN*-*D4* in wheat improvement and for our current efforts to clone this vernalization gene.

**Electronic supplementary material:**

The online version of this article (doi:10.1007/s00438-013-0788-y) contains supplementary material, which is available to authorized users.

## Introduction

Wheat (*Triticum aestivum* L.) is the second largest crop in dietary intake and the first in harvested area worldwide (FAO 2012), which makes it a critical resource for world food security. The wide distribution of wheat and its ability to grow in very different environments is attributed in part to the plasticity of the gene network that regulates its reproductive development (Distelfeld et al. 2009a). This plasticity allows wheat to adapt to very different growing conditions and is used by wheat breeders to maximize seed production under different or changing environments.

Photoperiod and temperature are the two main environmental cues used by wheat to adjust its flowering time to seasonal changes. Winter wheats, which are sown in the fall, require a prolonged exposure to cold temperatures (known as “vernalization”) to accelerate flowering. The vernalization requirement delays reproductive development, protecting the sensitive reproductive organs from frost damage during the winter.

In wheat and barley, the vernalization requirement is mainly controlled by four genes, *VRN1*, *VRN2*, *VRN3* and *VRN4* (Danyluk et al. 2003; Trevaskis et al. 2003; Yan et al. 2003, 2004a, 2006; Yoshida et al. 2010). The first three vernalization genes have been cloned and the mutations responsible for the loss of the vernalization requirement have been characterized (Yan et al. 2004b, 2006; Fu et al. 2005; von Zitzewitz et al. 2005; Distelfeld et al. 2009b; Nitcher et al. 2013). Homoeologs of *VRN1*, *VRN2*, and *VRN3* have been identified in the three genomes of hexaploid wheat (A, B and D genomes), but natural variation at the *VRN4* locus has been identified only in the D genome. In wheat, a letter is used before the gene number to indicate the genome and therefore, *VRN4* will be referred hereafter as *VRN*-*D4*.

*VRN1* encodes a MADS-Box transcription factor with high similarity to the meristem-identity gene *AP1* in Arabidopsis (Danyluk et al. 2003; Trevaskis et al. 2003; Yan et al. 2003). Natural variation at the *VRN1* promoter region or at the first intron is associated with a reduction or elimination of the vernalization requirement and with a relatively rapid upregulation of *VRN1* transcript levels in both apices and leaves of non-vernalized plants (Yan et al. 2004b; Fu et al. 2005). *VRN1* null mutants have a very late flowering but still produce normal flowers and fertile seeds (Chen and Dubcovsky 2012). The main function of the upregulation of *VRN1* in the leaves in the spring is to downregulate *VRN2* (Chen and Dubcovsky 2012).

*VRN2* is a long day (LD) flowering repressor with no clear ortholog in *Arabidopsis* (Yan et al. 2004a). The closest *VRN2* ortholog in rice, *Ghd7* (colinear with wheat *CONSTANS*-*like* *9*), is also a long day flowering repressor (Xue et al. 2008), but is not regulated by vernalization. The wheat *VRN2* locus includes two related paralogs designated *ZCCT1* and *ZCCT2* (Yan et al. 2004a), based on the presence of two putative zinc fingers and CCT domains (for *C* *O*, *C* *O*-*like*, *T* *OC1*). Simultaneous deletions or mutations of both *ZCCT1* and *ZCCT2* genes (in all homoeologs) are associated with recessive alleles for spring growth habit (Yan et al. 2004a; Distelfeld et al. 2009b; Hemming et al. 2009). The main role of *VRN2* is to repress the induction of *VRN3* in the fall, to prevent the induction of flowering before the winter.

*VRN3* is a close homolog to the *FLOWERING LOCUS T* in Arabidopsis, and is also referred to as *TaFT1* (Yan et al. 2006). In Arabidopsis, the FT protein is induced in the leaves by long days and is then transported to the apices where it interacts with FLOWERING LOCUS D (FD) and binds to the *AP1* promoter to induce flowering (Wigge et al. 2005). A similar result has been described in wheat, where the VRN3 protein interacts with FLOWERING LOCUS D-like 2 (FDL2), which has been shown to bind to the *VRN1* promoter in vitro (Li and Dubcovsky 2008). *VRN3* integrates signals from the photoperiod (*CO1*, *CO2* and *PPD*) and vernalization (*VRN1* and *VRN2*) pathways (Yan et al. 2006; Dubcovsky et al. 2006; Hemming et al. 2008, 2009). *VRN3* alleles with unusually high levels of expression are associated with dominant spring growth habit in both wheat (Yan et al. 2006) and barley (Nitcher et al. 2013). Before vernalization, *VRN3* transcript levels are maintained at low levels by *VRN2.* During vernalization, the upregulation of *VRN1* in the leaves results in the downregulation of *VRN2* and the release of *VRN3*, which can further upregulate *VRN1* in the leaves (then transported to the apices), generating a positive feedback regulatory loop that results in the irreversible induction of flowering (Loukoianov et al. 2005; Chen and Dubcovsky 2012).

*VRN*-*D4* has not been cloned so far and is not as well-characterized as the three vernalization genes described above. The dominant spring allele *Vrn*-*D4* is found mainly in hexaploid wheat varieties from Asia, especially in India and nearby regions (Iwaki et al. 2000, 2001). The *Vrn*-*D4* allele was transferred to Western wheat varieties from the Indian cultivar Muzaffarnagar into the cultivar Gabo, which was widely cultivated in Australia between the late 1940s and the late 1960s (O’Brien et al. 2001). Gabo was then backcrossed into Triple Dirk to develop an isogenic line named “TDF”, that is the designated genetic stock for the dominant *Vrn*-*D4* allele (Pugsley 1972). Incorrect stocks of TDF generated conflicting results on the effect of *Vrn*-*D4*, but it was recently confirmed that the stock maintained at Okayama University, Japan (designated TDF-J) has the correct *Vrn*-*D4* (Yoshida et al. 2010). A low-resolution genetic linkage map placed the *VRN*-*D4* gene on the centromeric region of chromosome 5D, within a 1.8 cM interval flanked by SSR markers *cfd78* on the short arm and *barc205* on the long arm, and far from *VRN*-*D1* which is located in the middle of the long arm (Yoshida et al. 2010).

The centromeric region of homoeologous group 5 includes the *TaVIL1* gene (Fu et al. 2007) which encodes a homolog of the Arabidopsis homodomain (PHD) finger protein VIL1 (VERNALIZATION INSENSITIVE 3(VIN3)-LIKE 1). This protein, together with VIN3, plays an important role in the epigenetic memory of vernalization and has an additional role in the photoperiodic regulation of flowering time in Arabidopsis (Sung et al. 2006). Based on its similarity with the Arabidopsis *VIL1*, its centromeric location and its transcriptional upregulation during vernalization, the wheat *TaVIL*-*D1* gene was previously considered as a potential candidate gene for *VRN*-*D4* (Fu et al. 2007).

In this study, we sequenced the wheat *TaVIL*-*D1* gene in lines carrying different *VRN*-*D4* alleles to evaluate its potential as a candidate gene for *VRN*-*D4*, and developed a high-density genetic map of the 5D centromeric region to precisely map both genes. We mapped *VRN*-*D4* into a 0.09 cM interval in the centromeric region of chromosome 5D, and identified the colinear regions in *Brachypodium distachyon* and rice. Since *VRN*-*D4* was completely linked to the centromere of chromosome 5D, we generated telocentric stocks 5DS and 5DL from TDF-J to further dissect this region. Finally, we characterized the epistatic interactions between *VRN*-*D4* and the other vernalization genes to better understand the role of *VRN*-*D4* in the wheat vernalization pathway.

## Materials and methods

### Plant materials

Near isogenic line Triple Dirk F was obtained from Okayama University, Japan (TDF-J, hereafter), and is the same line as the one used for the initial low-density mapping of *VRN*-*D4* on chromosome 5D (Yoshida et al. 2010). TDF-J has a spring growth habit determined by the dominant *Vrn*-*D4* allele and carries alleles for winter growth habit for all the homoeologs of the other three vernalization genes (*vrn1*, *Vrn2*, and *vrn3*).

TDF-J was crossed with a disomic substitution line of *Aegilops tauschii* chromosome 5D in Chinese Spring designated hereafter CS(5D_5402_). A segregating population of 1,591 F_2_ plants (3,182 gametes) was generated from this cross to construct the *VRN*-*D4* high-density map. CS(5D_5402_) was selected as a parental line because of its extensive polymorphism with *T. aestivum* chromosome 5D (http://wheat.pw.usda.gov/SNP/new/index.shtml). Wheat chromosome 5D has low genetic diversity (Akhunov et al. 2010) which makes its mapping exceedingly difficult. To develop CS(5D_5402_), synthetic wheat RL5402 was crossed as a male with CS monotelosomic 5DL, monosomic progeny was selected, and four times backcrossed to the monotelosomic, selecting a monosomic progeny in each backcross generation. Synthetic wheat RL5402 is a hexaploid amphiploid produced from a cross of tetraploid wheat ‘Tetra Canthatch’ with *Ae. tauschii* ssp. *tauschii* RL5261 (Kerber 1964; Kerber and Rowland 1974). CS(5D_5402_) has a winter growth habit, because the replacement of CS chromosome 5D by *Ae. tauschii* chromosome 5D resulted in the replacement of the *Vrn*-*D1* allele for spring growth habit by the *vrn*-*D1* allele for winter growth habit.

Chinese Spring nulli-tetrasomic lines for chromosome 5D and ditelosomic line Dt5DL carrying only the long arm of chromosome 5D (Endo and Gill 1996; Linkiewicz et al. 2004; Sears and Sears 1979) were employed to determine the chromosome and arm location of the markers used in this study. Arm locations were validated using DNAs extracted from flow-sorted 5DS and 5DL telocentric chromosomes from CS (Institute of Experimental Botany, Olomouc, Czech Republic).

To determine the arm location of *VRN*-*D4* telocentric chromosomes for 5D TDF-J were produced by centric misdivision of a 5D univalent generated from the cross between nulli-tetrasomic stock CSN5DT5B and TDF-J. The F_1_ plants were self-pollinated and 192 F_2_ plants were screened by C-banding for the presence of misdivision products using the methods described before (Lukaszewski and Gustafson 1983).

### Growth conditions

Plants were grown in a greenhouse under non-vernalizing temperatures (20–25 °C) and long day photoperiod (16 h light). Natural day length in the greenhouses was supplemented with incandescent lamps at night to extend the photoperiod to 16 h. Heading times were registered from sowing to spike emergence.

### Genetic and physical maps

Primers for the seven microsatellite markers included in the high-density map (*barc143*, *gdm3*, *wmc318*, *barc205*, *cfd67*, *cfd78* and *cfd81*) were obtained from the GrainGenes database (http://wheat.pw.usda.gov/GG2/). Primers, PCR conditions, restriction enzymes and size of the indel polymorphism for the 19 sequence-based markers developed in this study are listed in Table S1 (supplemental materials). These markers included cleavage amplified polymorphic sequences (CAPs) and sequence tag site (STS). Information for marker *BG313707* has been previously published (Yoshida et al. 2010). Markers were assigned to chromosomes and chromosome arms using the genetic stocks described above.

Genomic DNA was extracted from young leaves of individual plants using the cetyltrimethylammonium bromide (CTAB) method (Murray and Thompson 1980). PCR reactions were conducted in a total volume of 20 μl containing 10 mM Tris–HCl, PH 8.3, 50 mM KCl, 2 mM MgCl_2_, 0.2 mM dNTP, 0.5 μM of each primer, 50–100 ng of genomic DNA, and 0.5 U *Taq* DNA polymerase. PCR amplification was performed at 94 °C for 5 min, followed by 40 cycles of 94 °C for 30 s, annealing for 30 s, and 72 °C for 1 min/kb, with a final extension at 72 °C for 7 min.

Annealing temperatures for different microsatellite markers were as follows: 57 °C for *gdm68*, *cfd78* and *barc143*; 60 °C for *cfd67*, *gdm3* and *wmc318*; 65 °C for *bac205*, and 60 °C for marker *TaVIL1*. PCR products for markers *BE445181*, *BE499257*, *BE591275*, *BE606654*, *DQ512349* (=*TaAGL31*), *BE403761*, *BJ315664*, *CJ521028*, and *BE444702* were separated on 1 % agarose gels. All other sequence-based markers and SSR markers were separated on 6 % polyacrylamide gels. All gels were stained and visualized with 2 % ethidium bromide.

### Characterization of *TaVIL*-*D1* and other candidate genes

The initial *TaVIL1* sequences were obtained from *TmVIL*-*A* ^*m*^ *1* from *Triticum monococcum* (Fu et al., 2007) and from BAC clone WCS1202I10 (AB845602), selected from the Chinese Spring BAC library (Allouis et al. 2003) using a PCR marker for *TaVIL*-*D1*. Primers and PCR conditions used to re-sequence *TaVIL*-*D1* in the different genetic backgrounds are listed in Table S2. We first sequenced the *VIL1* gene from diploid wheat and *Aegilops* species *Ae. tauschii* (D genome, AB845601), *Ae. speltoides* (S genome closely related to the B genome of wheat, AB845599 and AB845600) and *Triticum urartu* (A genome, AB845597 and AB845598) to obtain sequences related to the A, B and D genomes of hexaploid wheat, respectively. Based on these sequences, we designed D-genome-specific primers and tested them in nulli-tetrasomic lines for homoeologous group 5. The D-genome specific primers were then used to sequence *TaVIL*-*D1* in hexaploid wheat lines TDF-J, CS(5D_5402_), Triple Dirk C (TDC), and two Japanese winter wheats lines (Hayakomugi and Akakawaaka). A similar strategy was used to retrieve the D-genome-specific sequences of other *VRN*-*D4* candidate genes.

The *TaVIL*-*D1* sequenced region including 1 kb upstream from the start codon and 1.2 kb downstream from the stop codon, was compared among TDF-J, TDC, Hayakomugi, Akakawaaka, and CS(5D_5402_), and polymorphisms were tested for association with differences in growth habit and *VRN*-*D4* alleles. A 12 bp indel polymorphism in *TaVIL*-*D1* exon 2 identified between TDF-J and CS(5D_5402_) was used to integrate *TaVIL*-*D1* into the high-density map (primers VIL1-D-F2 GTTGTTTCCTGTCCATACTAACGC, and VIL1-D-R2 GGCTTTTTGTCTTGAAACATTTT).

### Comparative genomics analysis and gene analysis

To delimit the region on *B. distachyon* that is colinear with the *VRN*-*D4* candidate region in wheat, sequences of the wheat Expressed Sequence Tags (ESTs) flanking *VRN*-*D4* were used as queries to search the *B. distachyon* genome (Brachy1.0 database, http://www.brachypodium.org/) using the BLASTN algorithm. The *B. distachyon* genes were then used to find the rice orthologs at the National Center for Biotechnology Information (NCBI, http://www.ncbi.nlm.nih.gov/blast/Blast.cgi) and Department of Energy’s Joint Genome Institute and the Center for Integrative Genomics website (http://www.phytozome.net). Since rice does not have a vernalization response and is evolutionarily less related to wheat compared to *B. distachyon*, we focused on the *B. distachyon* colinear region to search for potential *VRN*-*D4* candidate genes.

Conserved domains present in the *B. distachyon* proteins encoded by the genes present in the colinear candidate region were analyzed using the Conserved Domain Search at NCBI (http://www.ncbi.nlm.nih.gov/Structure/cdd/cdd.shtml) and InterPro Scan at EMBL-EBI (http://www.ebi.ac.uk/Tools/pfa/iprscan/).

Based on this information and on the annotation of these genes in other species, we identified transcription factors and genes known to be involved in development. We searched for the closest wheat homologs in wheat NCBI databases, genomic sequences from flow-sorted wheat chromosomes arms developed by the International Wheat Genome Sequencing consortium (IWGSC, http://urgi.versailles.inra.fr), and recent wheat transcriptomes (Krasileva et al. (2013), http://maswheat.ucdavis.edu/Transcriptome/). We then did a reverse BLASTP of the best wheat candidate to the *B. distachyon* proteome to confirm that the correct homolog was found.

### Expression analysis of candidate genes by qRT-PCR

RNA samples were extracted from leaves using the Spectrum Plant Total RNA Kit (Sigma-Aldrich). Purified RNA samples were checked for RNA integrity by running 0.5 μg RNA on a 1 % agarose gel. All samples showed clear 18S and 26S ribosomal RNA bands indicating lack of RNA degradation. Forward primers for the qRT-PCR (CCAGATGCTGCAAAGCACTA and GAGCTCATTCTTCCTGGCC, efficiency 91 %) were designed in conserved regions of the A-, B- and D-genome copies of *TaAGL31*, the reverse primer was designed to anneal over a conserved exon-junction site to avoid amplification on genomic DNA. Melting curves showed a single peak, which confirmed amplification of a single product. Standard curves were constructed to calculate amplification efficiency for the SYBR Green^®^ system developed for *TaAGL31*. Quantitative PCR was performed using SYBR Green^®^ and a 7500 Fast Real-Time PCR system (Applied Biosystems). *ACTIN* was used as an endogenous control using primers described before (Uauy et al. 2006). Transcript levels are expressed as linearized fold-*ACTIN* levels calculated by the formula $2^{{\left( {ACTIN\;{\text{C}}_{\text{T}} {-}TARGET\;{\text{C}}_{\text{T}} } \right)}}$.

### Epistatic interactions

To evaluate the epistatic interactions among *VRN1* and the other known vernalization genes, we developed four different segregating populations. The TDF-J line was crossed with Triple Dirk near isogenic lines (NILs) TDD, TDB and TDE carrying the *Vrn*-*A1*, *Vrn*-*B1* and *Vrn*-*D1* dominants alleles, respectively [described by Yoshida et al. (2010)]. An additional F_2_ population was developed from the cross between TDF-J and CS(Hope7B) chromosome substitution line (Yan et al. 2006) to test the epistatic interactions between *Vrn*-*D4* and *Vrn*-*B3* (=*FT*-*B1*). The Hope 7B chromosome introgressed into CS carries a highly expressed *Vrn*-*B3* allele with a repetitive element inserted in the promoter region of *FT* (Yan et al. 2006). Approximately, 100 plants per F_2_ population were planted in a greenhouse (all four populations in the same experiment) under non-vernalizing (≥24 °C) and long day (16 h light) conditions. The *Vrn*-*A1*, *B1*, *D1* and *Vrn*-*B3* genotypes of the F_2_ plants were determined by PCR using primers described before (Fu et al. 2005; Yan et al. 2006). The *VRN*-*D4* genotype was inferred using SSR marker *barc205* located 1.2 cM distal from *VRN*-*D4* (Yoshida et al. 2010).

The number of days between planting and complete emergence of the first spike was scored for each F_2_ plant. The data was evaluated in three 2 × 3 factorial ANOVA considering *VRN*-*D4* and each of the other vernalization loci as factors with three levels: homozygous *Vrn*-*D4,* heterozygous, and homozygous *vrn*-*D4*. In the case *Vrn*-*B3* × *Vrn*-*D4*, an additional gene (*Vrn*-*D1*) segregates in the F_2_ population, so only a subpopulation of 33 plants carrying the winter allele for *Vrn*-*D1* was tested for epistatic interactions between *Vrn*-*B3* and *Vrn*-*D4* genes. Statistical analyses were performed using SAS version 9.3. Adjusted LS means are reported with their respective standard errors (SE).

The degree of dominance was calculated using the formula: $D = \left( {2X_{2} - X_{1} - X_{3} } \right)/\left( {X_{1} - X_{3} } \right)$ (Falconer 1960) where *X*_1_, *X*_2_ and *X*_3_ are the heading time (HT) values of, respectively, the plants homozygous for the late flowering allele (*vrn*), the heterozygous, and the plants homozygous for the early flowering allele (*Vrn*).

We also compared the effect of *Vrn*-*D4* relative to other vernalization genes (*VrnX*) on heading time (HT) when each gene was present in heterozygous state in a winter genetic background using the formula: ${\text{Relative single dose effect}} = {\text{HT}}\left( {VrnD4/vrnD4 \, vrnX/vrnX} \right) - {\text{HT}}\left( {VrnX/vrnX \, vrnD4/vrnD4} \right)$.

## Results

### Sequencing of *TaVIL*-*D1* candidate gene

The *TaVIL*-*D1* gene region was sequenced in TDF-J (AB846583) and five lines not carrying the *Vrn*-*D4* allele for early flowering: Hayakomugi (AB846584), CS(5D_5402_) (AB846585), TDC (AB846586), Akakawaka (AB846587) and *Ae. tauschii* (AB845601). The sequenced regions extended from 1 kb upstream of the start codon to 1.2 kb downstream of the stop codon, providing a total coverage of 5.4 kb. All these lines, with exception of TDF-J, have a winter growth habit.

The only polymorphisms found in these sequences were between the lines carrying the 5D chromosome from *T. aestivum* and the ones carrying the 5D chromosome from *Ae. tauschii* [CS(5D_5402_) and *Ae. tauschii* differ in a single SNP in the fourth exon]. CS(5D_5402_) has 18 SNPs, four deletions and one insertion compared with the 5D sequences from the *T. aestivum* lines. Among the six SNPs present in the exons, five were associated with non-synonymous substitutions. In addition, we found a 12 bp deletion in exon 2 that resulted in the deletion of four amino acids in CS(5D_5402_), but all were located outside of the PHD finger, FNIII, or VID conserved domains.

We found no polymorphisms in the *TaVIL*-*D1* coding regions or in the 5′ (1.0 kb) and 3′ (1.2 kb) regions between the spring TDF-J (*Vrn*-*D4* allele) and the three *T. aestivum* lines with winter growth habit (Hayakomugi, TDC, and Akakawaaka, *vrn*-*D4* allele). The sequencing data suggests that *TaVIL*-*D1* is an unlikely candidate for *VRN*-*D4*, but we could not rule out the possibility of a polymorphic regulatory sequence outside the sequenced region. To provide more conclusive evidence, we developed a high-density mapping population from the cross TDF-J × CS(5D_5402_) and screened it for recombination events within the *VRN*-*D4* region, which includes the *TaVIL*-*D1* gene.

### High-density map of *VRN*-*D4*

**Fig. 1:**
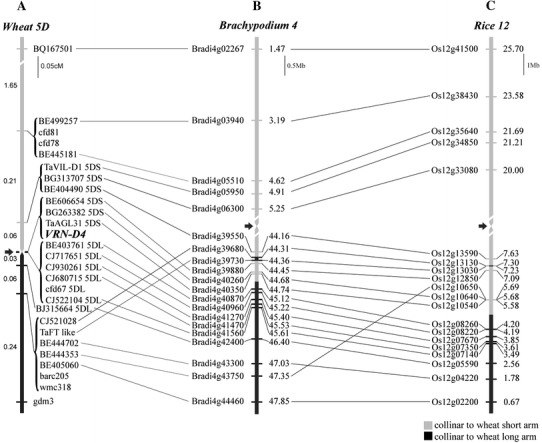
High-density comparative map of the *VRN*-*D4* candidate region. **a** High-density genetic map of the *VRN*-*D4* in hexaploid wheat cross TDF-J × CS(5D_5402_). Distances are in cM. **b, c** Maps of *B. distachyon* and rice colinear regions with *VRN*-*D4* region. Distances are in Mb and are based on current genome sequences. *Arrows* indicate active centromeres, centromeric regions are not drawn to scale. **b** *B. distachyon* chromosome 4. **c** Rice chromosome 12

We mapped the *TaVIL*-*D1* gene close to the centromeric region of wheat chromosome arm 5DS. We also detected homoeologs of *TaVIL*-*D1* in the sequences of the 5AS and 5BS sorted chromosome arms sequenced by the IWGSC (http://wheat-urgi.versailles.inra.fr/Seq-Repository/BLAST), and in the sequences of barley chromosome arm 5HS, suggesting a conserved position in the *Triticeae.* Orthologs of *TaVIL*-*D1* were found in the colinear regions of *B. distachyon* chromosome 4 (*Brad4g05950*, 4.91 Mb) and rice chromosome 12 (*Os12g34850*, 21.21 Mb) (Fig. 1).

In spite of the limited recombination observed in the centromeric region of chromosome 5D in this segregating population (Yoshida et al. 2010), the expansion of the mapping population to 1,591 F_2_ plants (3,182 gametes) yielded two independent recombination events between *TaVIL*-*D1* and *VRN*-*D4* (Table 1, plant 189 and 768). Heading times for the progeny tests of these two critical plants (bottom row of Table 1) demonstrated that they were heterozygous for *Vrn*-*D4* but homozygous for *TaVIL*-*D1*. These recombination events, together with the lack of *TaVIL*-*D1* polymorphisms between TDF-J (*Vrn*-*D4* allele) and both TDC and the two hexaploid Japanese wheat cultivars (*vrn*-*D4* allele), indicate that the wheat *TaVIL*-*D1* gene is a very unlikely candidate gene for *VRN*-*D4*.

**Table 1:**
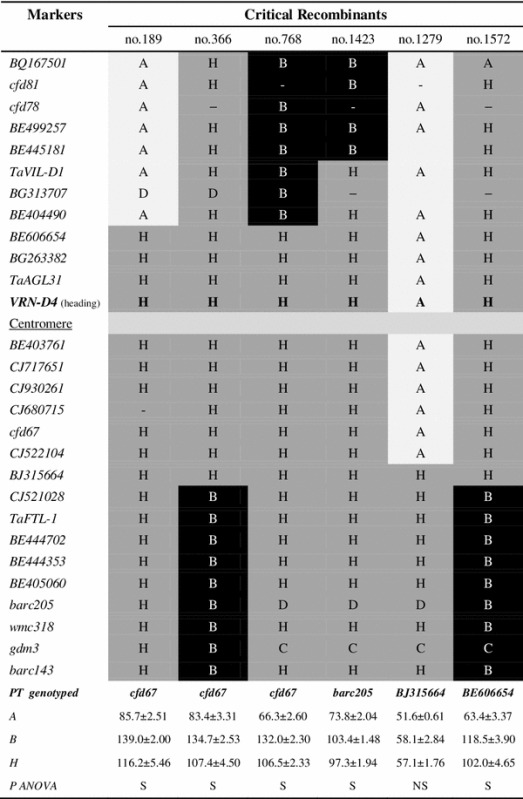
Graphical representation of haplotypes in the critical recombinants

A change in cell color indicates a recombination event. Heading time of each genotypic class is shown as mean ± standard error of the mean at the bottom of the Table (the heterozygous marker used to genotype the progeny test (PT) is indicated above)

*A* TDF-J, *B* CS(5D_5402_), *H* heterozygous, *C* B or H, *D* A or H, *S* significant (*P* < 0.0001), *NS* on-significant

To further dissect the *VRN*-*D4* region, we developed nineteen new sequence-based molecular markers in the centromeric region of chromosome 5D using wheat ESTs with significant sequence similarity to rice and *B. distachyon* genes located in the colinear regions (Fig. 1). Using these new markers and the new recombinant chromosomes described in Table 1, we mapped *VRN*-*D4* within a 0.09 cM interval flanked by markers *BE404490* and *BJ315664* in the centromeric region of chromosome 5D.

The nine sequence-based markers completely linked to the centromere could not be ordered based on recombination, but were divided into two groups by their arm location. The gene-based markers within these subgroups were ordered on the basis of the orthologous genes in *B. distachyon* (Fig. 1). To determine the most likely position of SSR marker *cfd67* within the 5DL subgroup, we first identified 5D scaffold 72311 as the closest match between the *cfd67* sequence and the *Ae. tauschii* genome (Jia et al. 2013). We then identified a gene within this scaffold and identified the *B. distachyon* orthologous gene as *Bradi4g41527*. Since this *B. distachyon* gene is located between *Bradi4g41470* and *Bradi4g41560* on chromosome 4 (45.59 Mb), *cfd67* was placed between the orthologous wheat genes *CJ680715* and *CJ522104*.

*VRN*-*D4* co-segregated with three markers mapped on the short arm and six markers mapped on the long arm of chromosome 5D and, therefore, could not be assigned to a specific chromosome arm. To obtain this information, we produced telocentric lines of chromosome 5D from TDF-J.

### Mapping of *VRN*-*D4* using monotelosomic and ditelosomic lines of TDF-J

**Fig. 2:**
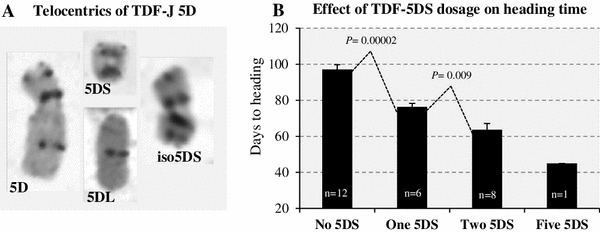
Use of ditelocentric TDF-J lines to map *VRN*-*D4.* **a** C-Banding of chromosome 5D, telocentric chromosomes 5DS and 5DL and isochromosome iso5D. **b** Heading time of lines carrying different dosages of the 5DS arms: 0 copies (mt5DL, dt5DL and nullisomics 5D), one copy (mt5DS), two copies (dt5DS and TDF-J), and five copies (dt-iso5DS + 5D). The number of plants in each class is indicated at the base of the bar and the standard error of the mean in the error bars. The *P* values are the results of two *t* tests for samples with unequal variances on data transformed to restored normality of residuals (Shapiro–Wilk test not significant). The dt-iso-5DS + 5D line was not compared statistically because a single plant was obtained in this class. Note the acceleration of flowering with increase dosage of the 5DS chromosome arm

To identify 5DS and 5DL monotelosomic chromosomes, we screened 192 F_2_ plants for the presence of misdivision products of chromosome 5D from TDF-J by C-banding. We identified two monotelosomic 5DL plants (mt5DL), one monotelosomic 5DS plant (mt5DS) and three plants with a normal 5D chromosome plus one isochromosome 5DS (iso5DS indicates two short arms fused at the centromere) (Fig. 2a).

These plants were grown, self-pollinated and their progenies were screened for the presence or absence of the telocentrics/isochromosomes and for their heading time (Fig. 2b). Since the *Vrn*-*D4* allele from TDF-J is partially dominant for early flowering (Yoshida et al. 2010), the lines missing the arm harboring *Vrn*-*D4* should show delayed heading time. The 12 plants missing the 5DS arm (three mt5DL, four dt5DL and five nullisomics 5D) flowered 97.1 ± 2.6 days after sowing, which is 20.8 days later than the average of the mt5DS plants carrying one copy of the 5DS chromosome (76.3 ± 1.9, *P* = 0.00002). This result indicates that *VRN*-*D4* is located in the short arm of chromosome 5D. This chromosome location was further confirmed by the comparison between the mt5DS and the combined dt5DS and TDF-J plants, which carry two doses of chromosome arm 5DS and flowered 12.7 days earlier (63.6 ± 3.5, *P* = 0.009) than the plants carrying one dose. No statistical comparison was made with the di-iso5DS + 5D (5 doses of 5DS), because only a single plant was recovered with this genotype. However, it is still interesting to point out that this was, by far, the earliest flowering line (45 days). Taken, together the data from this experiment consistently indicate that *VRN*-*D4* is located in the short arm of chromosome 5D.

### *VRN*-*D4* candidate genes in *B.distachyon* colinear region

The DNA sequences of the markers mapped on the centromeric region of chromosome 5D were used to delimit the colinear regions in *B. distachyon* genome (http://www.brachypodium.org) and in rice (http://www.phytozome.net). The 7 bins including the 20 wheat sequence-based markers (Fig. 1) showed good colinearity with *B. distachyon*, except for loci *CJ521028* and *TaFT*-*like* (we do not know the order of the wheat markers completely linked within a bin). All these *B. distachyon* genes, except one (*Bradi4g43750*), were colinear between *B. distachyon* and rice. In spite of the good colinearity observed in this region, the centromere of wheat chromosome 5D was mapped in a non-colinear position. Using the Chinese Spring ditelocentric 5DL chromosome, the wheat 5D centromere was mapped between markers *TaAGL31* and *BE403761*, 0.06 cM proximal to the position of the centromere predicted by rice and *B. distachyon* (Fig. 1).The closest markers flanking the wheat 5D centromere are colinear with a region in *B. distachyon* chromosome 4 (44.74–45.12 Mb) that is 22 Mb apart from the active *B. distachyon* centromere (22.5–22.9 Mb, Qi et al. 2010). The same wheat markers are colinear with a region in rice chromosome 12 (4.21–5.58 Mb) that is 6.4 Mb apart from the location of the rice centromere (12 Mb, Qi et al. 2010).

We then looked for putative candidate genes for *VRN*-*D4* in the colinear region of the sequenced *B. distachyon* genome (closer to wheat than rice). Wheat genes flanking *VRN*-*D4*, BE404490 on the short arm and BE403761, the most proximal marker in the long arm (Fig. 1 and Linkiewicz et al.^2004^), were used to delimit the colinear region on *B. distachyon* chromosome 4 between orthologous genes *Bradi4g39550* and *Bradi4g40870*. The selected *B. distachyon* region is roughly 1 Mb (44.16–45.13 Mb) and includes 127 predicted genes (http://www.brachypodium.org). Among these genes, ten have been annotated as transcription factors or developmentally related genes (Table 2) and are discussed in detail below.

**Table 2:** Selected putative candidate genes annotated as transcription factors or with functions in development

*B. distachyon* gene	Rice gene	Conserved protein domains [NCBI conserved domain id]	Top Arabidopsis BLASTP hit NCBI accession number and *E* value	Wheat orthologous (URGI contig ID)
Bradi4g39630	Os12g13170	B_zip[cd12193], basic leucine zipper DNA-binding and multimerization region of GCN4 and related proteins; MFMR[pfam07777], G-box binding protein MFMR	NP_850248.2; Basic region/leucine zipper transcription factor 16; *E* = 1e^−120^	IWGSC_chr5DL_ab_k71_4513163
Bradi4g40057	Not Found	Zinc finger, PHD-type (IPR001965), Zinc finger, RING/FYVE/PHD-type	NP_200350; Enhanced downy mildew; *E* = 2e^−63^	IWGSC_chr5DL_ab_k71_4518453
Bradi4g40230	Not colinear	DUF3594 super family [cl13583], RING super family [cl15348], RING-finger domain, specialized type of Zn-finger	XP_002875129.1; PHD finger family protein; *E* = 2e^−89^	Not found
Bradi4g40250	Os12g10660	BBOX super family [cl00034], B-Box-type zinc finger; zinc binding domain (CHC3H2); 3.56e-04	XP_002887747.1; zinc finger (B-box type) family protein; *E* = 3e^−38^	IWGSC_chr6DS_ab_k71_2071445
Bradi4g40270	Os12g10630	ZF-HD_dimer super family [cl04737]. homeo_ZF_HD super family[cl11752], homeobox domain, ZF-HD class	NP_172896.1; AtHB31; DNA binding/transcription factor (AtHB31); *E* = 3e^−48^	Not found
Bradi4g40310	Not colinear	zf-TRAF super family[cl08341], TRAF-type zinc finger	NP_187801.3; Prenyltransferase/zinc ion binding; *E* = 2e^−84^	IWGSC_chr7DL_ab_k71_ 3381171
Bradi4g40350	Os12g10540	MADS-box [cd00265] Type II subfamily of MADS. K-box super family [cl03234], K-box region	CAA16753.1; floral homeotic protein agamous; *E* = 2e^−67^	IWGSC_chr5DS_ab_k71_2764238 (= TaAGL31 = DQ512349)
Bradi4g40357	Os12g10520	MADS-box [cd00265] Type II subfamily of MADS. K-box super family [cl03234], K-box region	AAC49085.1; MADS-box protein AGL12; *E* = 2e^−47^	Not found
Bradi4g40540	Os12g09250	BRLZ[smart00338], basic region leucin zipper	XP_002888232; predicted protein; *E* = 1e^−68^	IWGSC_chr5BS_ab_k71_2249326 (Pseudogene, not in 5AS & 5DS)
Bradi4g40517	Os12g09590	RRM_like_XS[cd12266], RNA recognition motif-like XS domain found in plants; zf-XS super family[cl04095], XS zinc finger domain	XP_002872063; hypothetical protein ARALYDRAFT_489218; *E* = 1e^−167^	IWGSC_chr5DL_ab_k71_4509178

Only genes present in the regions of *B. distachyon* chromosome 4 and rice chromosome 12 regions colinear to the wheat *VRN*-*D4* region are presented

Three of the selected *B. distachyon* genes in Table 2 (*Bradi4g39630*, *Bradi4g40057*, and *Bradi4g40517*) showed close homology with wheat genes located in the long arm of chromosome 5D and therefore, were discarded as *VRN*-*D4* candidate genes. This is an expected result, since one of the wheat flanking markers (*BE403761*) was selected on the long arm to guarantee the inclusion of all the short arm genes. For two of the other *B. distachyon* genes (*Bradi4g40250* and *Bradi4g40310*), the wheat genes with the lowest E values were located on wheat chromosome arms 6DS and 7DL, respectively (Table 2). As a result these two genes were also discarded as potential candidates for *VRN*-*D4.*

No wheat orthologs were found for another three *B. distachyon* genes in Table 2 (*Bradi4g40230*, *Bradi4g40270*, and *Bradi4g40357*) in any of the searched databases (see “Materials and methods”). Reverse BLAST of the wheat sequences with the lowest E values back to the *B. distachyon* genome identified a different *B. distachyon* gene from the one used in the original query. These results indicate that the detected wheat genes were paralogs rather than orthologs of these three *B. distachyon* genes. Interestingly, the reverse BLAST of the closest wheat sequences for *Bradi4g40230* and *Bradi4g40270* (*AK333859* and *UCW_Tt*-*k61_contig_71361*, 81 and 86 % identity at the DNA level, respectively) detected two close paralogs (*Bradi4g21040* and *Bradi4g21160*) mapping to the same centromeric region of *B. distachyon* chromosome 4. This region is colinear with the centromeric regions of wheat chromosome 4 and rice chromosome 11, and may represent an ancestral duplication. Rice chromosomes 11 and 12 are known to be the result of an ancient duplication (Paterson et al. 2004;  Wu et al. 1998).

*Brachypodium distachyon* gene *Bradi4g40540* showed homology with a sequence on wheat chromosome 5BS (IWGSC_chr5BS_ab_k71_2249326) but no significant similarity was detected with sequences on chromosome arms 5AS or 5DS in the IWGSC database (http://wheat-urgi.versailles.inra.fr/Seq-Repository). Annotation of the 5BS genomic sequence revealed the presence of a premature stop codon in the first intron, demonstrating that this sequence corresponds to a pseudogene and therefore, is not a valid candidate for *VRN*-*D4*.

The most promising candidate gene for *VRN*-*D4* was the *B. distachyon* gene *Bradi4g40350*, which showed 90 % identity with the wheat gene *TaAGAMOUS*-*like 31* (*TaAGL31* = *DQ512349*), a MADS-box gene related to the floral homeotic gene *AGAMOUS* in Arabidopsis (Table 2). *TaAGL31* is 100 % identical to a sequence detected on chromosome arm 5DS (IWGSC_chr5DS_ab_k71_2764238), the same arm location as *VRN*-*D4.* A reverse BLAST confirmed that *Bradi4g40350* is the closest *B. distachyon* gene to this wheat sequence. We sequenced *TaAGL31* in the two parental lines of our mapping population [TDF-J and CS(5D_5402_)] and used one of the detected SNPs to map this gene. *TaAGL31* was mapped completely linked to *VRN*-*D4* (Fig. 1; Table 1). We also sequenced the promoter (1,404 bp from the start codon), the first intron, the coding region and the 3′ UTR (438-bp) of this gene in the winter *T. aestivum* line TDC (*vrn*-*D4*) and found no polymorphisms with TDF-J (*Vrn*-*D4*) (KF761670 and KF761668 respectively). The *TaAGL31* coding region in these two accessions (KF761667, KF761666) was 100 % identical to the Chinese Spring sequence (DQ512349). Finally, we checked the expression of this gene in TDF-J and TDC by qRT-PCR at two different time points (3- and 5-week-old plants, Fig. 4), and found no differences in *TaAGL31* transcript levels between these two lines.

### Epistatic interaction of *VRN*-*D4* with vernalization genes

**Fig. 3:**
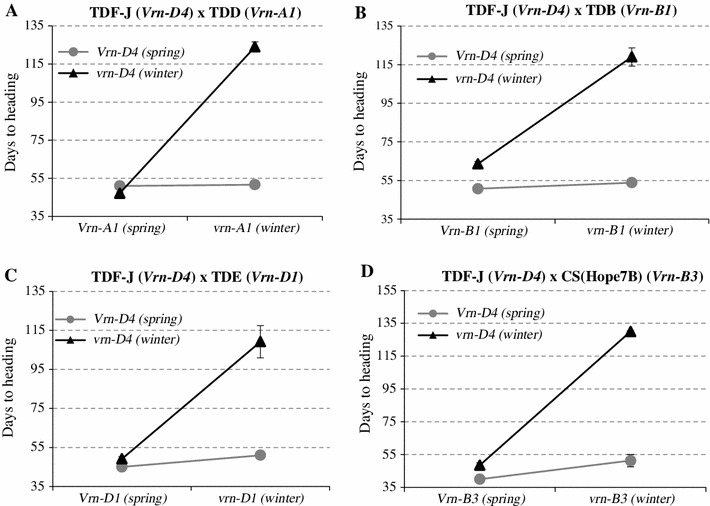
Interactions between *VRN*-*D4* and other vernalization genes. To facilitate the visualization of the interactions we only included in this figure the plants homozygous for the two vernalization genes segregating in each population. **a** Interactions between *VRN*-*D4* and *VRN*-*A1.* **b** Interactions between *VRN*-*D4* and *VRN*-*B1.* **c** Interactions between *VRN*-*D4* and *VRN*-*D1.* **d** Interactions between *VRN*-*D4* and *VRN*-*B3.* All the interactions were highly significant (*P* < 0.0001, based on 2 × 3 factorial ANOVAs including homozygous and heterozygous classes for each gene, Table 3)

The results of the 2 × 3 factorial ANOVAs for the four different segregating populations used to test the epistatic interactions between *VRN*-*D4* and other vernalization genes are presented in Table 3. The main effects of *VRN*-*D4*, *VRN1* three homologs (*VRN*-*A1*, *VRN*-*B1* and *VRND1*), and *VRN*-*B3* were highly significant (*P* < 0.0001) in all four populations. The interactions between *VRN*-*D4* and the other vernalization genes were also highly significant (*P* < 0.0001, Fig. 3a–d). In all four cases, *VRN*-*D4* showed a smaller effect on heading time when the early flowering allele of the other vernalization gene was present than when it was absent (Fig. 3a–d). All plants homozygous for the *vrn*-*D4* and *vrn*-*1* alleles for winter growth habit segregating in each population showed very late flowering (adjusted means average between 119 and 130 days, Fig. 3a–d), confirming that there were no additional genes segregating for spring growth habit in these populations. This was also supported by the large proportion of variation in heading time (*R* ^2^ = 0.87–0.96) explained in each population by the factorial ANOVA models including the two vernalization genes and their respective interactions.

**Table 3:** Interactions between *VRN*-*D4* and other vernalization genes

*Vrn*x	*P VrnX*	*P Vrn*-*D4*	*P* interaction	*R* ^2^	*VrnX* dominance^a^	*Vrn*-*D4* dominance^a^	*Vrn*-*D4* –*VrnX* one dose effect^b^
*Vrn*-*A1*	<0.0001	<0.0001	<0.0001	0.96	95.9 %	53.7 %	18.5 days***
*Vrn*-*B1*	<0.0001	<0.0001	<0.0001	0.91	55.6 %	30.7 %	1.0 days^NS^
*Vrn*-*D1*	<0.0001	<0.0001	<0.0001	0.89	78.2 %	42.9 %	14.3 days***
*Vrn*-*B3*	<0.0001	<0.0001	<0.0001	0.87	71.0 %	31.3 %	18.9 days**

Probability of main effects and interactions in the 2 × 3 factorial ANOVA, proportion of variation explained by the model (*R* ^2^) of the model, degree of dominance and relative effect of single dose allele combination

^a^Degree of dominance was calculated using LS Means and the formula described in “Materials and methods”

^b^Relative single dose effect was calculated using LS Means and the formula described in “Materials and methods”. Positive values indicate that plans carrying the *Vrn*-*D4* allele flowered later than plants carrying the *VrnX* allele

** *P* < 0.001, *** *P* < 0.0001 and ^NS^ *P* > 0.05

The degree of dominance (D) for the early flowering allele of *Vrn*-*D4* (30.7–53.7 %) was smaller than the degree of dominance of the different *Vrn1* alleles (55.6–95.9 %, Table 3). Among the *VRN1* genes, the *Vrn*-*A1* allele for spring growth habit showed the highest level of dominance, which is consistent with previous studies showing that a single dominant *Vrn*-*A1* allele is usually sufficient to eliminate the vernalization requirement.

We also showed that the *Vrn*-*D4* allele had a weaker effect to accelerate flowering than the *Vrn*-*A1*, *Vrn*-*D1* or *Vrn*-*B3* alleles. In genetic backgrounds where all other genes were homozygous for a winter growth habit, plants heterozygous only for *Vrn*-*D4* flowered 14–19 days later than those heterozygous only for *Vrn*-*A1*, *Vrn*-*D1* or *Vrn*-*B3* (Table 3). No significant differences were detected between *Vrn*-*D4* and *Vrn*-*B1*, which together with the similar dominance values of these two genes suggests that the dominant *Vrn*-*D4* allele has an effect on heading time approximately similar to that of the dominant *Vrn*-*B1* allele (Table 3).

## Discussion

### *VRN*-*D4* mapping

In the high-density map presented in this study, we were able to separate *VRN*-*D4* from four of the five markers that were completely linked to *VRN*-*D4* in previous maps (Yoshida et al. 2010), confirming higher resolution of this new map. In addition to the larger number of recombination events, the new map includes 19 new sequence-based markers in the centromeric region of chromosome 5D (short and long arms), which facilitated detailed comparisons with the colinear regions in the sequenced *B. distachyon* and rice genomes.

The high-density map of *VRN*-*D4*, and eventually its positional cloning, faces two major challenges. The first one is the limited polymorphisms in the *T. aestivum* D genome (Dubcovsky and Dvorak 2007; Akhunov et al. 2010), particularly in the centromeric regions. The second one is the limited recombination in the proximal half of the wheat chromosomes (Luo et al. 2013; Lukaszewski and Curtis 1993). The first problem was addressed in this study by studying recombination between homologous chromosomes 5D from *T. aestivum* and a divergent *Ae. tauschii* accession. These two chromosomes showed 120 SNP in 24.4 kb sequenced (~5 SNP/kb) compared with no SNPs detected in the 37.0 kb sequenced in chromosome 5D between TDF-J and both TDC and Hayakomugi.

Unfortunately, the higher level of polymorphisms in this population is associated with a ~30 % reduction in recombination frequency (Yoshida et al. 2010). This reduced recombination is possibly associated with the ability of the *Pairing homoeologous* (*Ph1*) gene (Yousafzai et al. 2010; Griffiths et al. 2006) to detect the relatively high level of heterozygosity between the *T. aestivum* and *Ae. tauschii* 5D chromosomes. The *Ph1* gene is known to produce a more drastic reduction in recombination between the A genome of wheat and the A^m^ genome from *T. monococcum* (Dubcovsky et al. 1995), which show an average of ~12 SNP/kb in intronic regions (Dubcovsky and Dvorak 2007). To solve the problem of the reduced recombination, we are developing new mapping populations in the *ph1b* mutant background segregating for the same 5D chromosomes from *T. aestivum* and *Ae. tauschii* used in this study [TDF-J × CS(5D_5402_)]. However, even if recombination is restored to levels observed in normal *T. aestivum* × *T. aestivum* crosses, the natural low level of recombination near the centromeric regions of wheat chromosomes will continue to complicate efforts to dissect the *VRN*-*D4* region by recombination. We are currently exploring the use of radiation mutants to generate additional break points in the 5D centromeric region.

### Comparative maps of the *VRN*-*D4* candidate region in *B. distachyon* and rice

The previous genetic map of *VRN*-*D4* was mainly based on SSR markers (only one sequence-based marker), limiting the comparisons to sequenced genomes from other grass species (Yoshida et al. 2010). The 20 sequence-based markers on the 5D centromeric region were mapped to 7 bins that show good colinearity with *B. distachyon* chromosome 4 and rice chromosome 12 (Fig. 1). Most of these *B. distachyon* loci were also colinear with rice, with the exception of *Bradi4g43750* (Fig. 1). Two closely linked wheat genes, *CJ521028* and *FT*-*like*, were not colinear with *B. distachyon* or rice (Fig. 1). The orthologs of these two genes are 0.06 Mb apart on *B. distachyon* chromosome 4 and do not seem to be physically close in wheat since they are located on non-overlapping contigs generated in the different wheat genomic projects (http://wheat-urgi.versailles.inra.fr/Seq-Repository/BLAST; Jia et al. 2013; Ling et al. 2013).

An interesting exception to the relatively good colinearity observed among wheat, *B. distachyon* and rice chromosomes in the *VRN*-*D4* region is the different position of the centromeres. In wheat, we mapped the centromere of chromosome 5D between markers *TaAGL31* on the short arm and BE403761 on the long arm. These two markers are completely linked genetically but were separated by the centric breakage in telocentric 5DS and 5DL, and by the use of DNAs from sorted 5DS and 5DL chromosome arms. BE403761 homoeologs have been also mapped in the centromeric region of the long arms on telocentric chromosomes 5BL and 5HL and in two translocations between the short arm of rye 5RS and the long arms of chromosomes 5B and 5D (T5RS.5BL and T5RS.5DL), suggesting conserved break points (Linkiewicz et al. 2004). The markers flanking the centromere on wheat chromosome 5D are colinear with *B. distachyon* markers *Bradi4g40350* and *Bradi4g40870* that delimit a region of chromosome 4 between 44.74 and 45.12 Mb. This region is more than 20 Mb distal from the location of the active centromere on *B. distachyon* chromosome 4 [22–23 Mb, Qi et al. (2010)]. Furthermore, pericentromeric probes for wheat chromosome 5 were mapped on the long arm of *B. distachyon* chromosome 4 at positions 44–46 Mb (Qi et al. 2010). This is the same region delimited by *Bradi4g40350* and *Bradi4g40870*, the markers colinear with the wheat 5D centromere. This region appears to be the location of an inactive *B. distachyon* centromere homologous to the active 5D centromere. On the other hand, probes from wheat chromosome 4 hybridized to the 22.5 Mb midpoint of *B. distachyon* chromosome 4, the expected position of the centromere in this metacentric chromosome (Qi et al. 2010). The sequencing of the *B. distachyon* genome confirmed that the active centromere of *B. distachyon* chromosome 4 is homologous to centromeres of wheat chromosome 4 and rice chromosome 11 (International Brachypodium Initiative 2010).

Rice chromosomes 11 and 12 are the result of an ancient duplication that predates the divergence of the major grass subfamilies (Paterson et al. 2004). The functional centromere of rice chromosome 12 has been mapped at 12 Mb, far from the region colinear with wheat 5D centromere, which is delimited by markers *Os12g10540* (5.58 Mb) and *Os12g08260* (4.20 Mb) (Fig. 1). In summary, these results indicate that the wheat 5D centromere is at a different location than the centromeres of rice chromosome 12 and *B. distachyon* chromosome 4.

### *VRN*-*D4* candidate region

*TaVIL*-*D1* has been previously suggested as a candidate for *VRN*-*D4* based on its centromeric location, homology with an Arabidopsis vernalization gene, and upregulation during vernalization (Fu et al. 2007). Results from the high-density map conclusively show that *TaVIL*-*D1* is not *VRN*-*D4*. Two independent recombination events were detected between the two loci, demonstrating that *TaVIL*-*D1* is proximal to *VRN*-*D4.* Further supporting this conclusion, we found no polymorphism in the coding or regulatory regions of *TaVIL*-*D1* between wheat accessions differing in *VRN*-*D4.* We are currently generating TILLING mutants of *TaVIL1* to study its function in wheat.

**Fig. 4:**
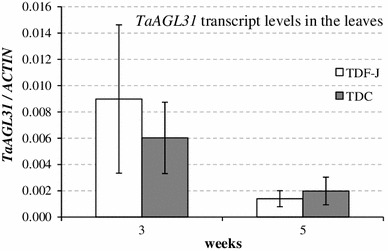
Comparison of *TaAGL31* transcript levels in the leaves of TDF-J (*Vrn*-*D4*) and TDC (*vrn*-*D4*) at two different time points. Transcript levels are expressed as linearized fold-*ACTIN* levels calculated by the formula $2^{{\left( {ACTIN\;{\text{C}}_{\text{T}} {-}TARGET\;{\text{C}}_{\text{T}} } \right)}}$. *Error bars* are SE of the means calculated from six biological replications

To look for alternative candidate genes, we explored the *VRN*-*D4* colinear region in *B. distachyon* (The International *Brachypodium* Initiative 2010). This species was selected among those with completely sequenced genomes based on its closer evolutionary relationship with wheat (Draper et al. 2001). The *VRN*-*D4* colinear region in *B. distachyon* chromosome 4 is approximately 1-Mb and includes a total of 127 predicted genes. Most of these genes have been annotated as structural or metabolic proteins and are unlikely candidates for *VRN*-*D4*. However, ten genes in this region were annotated as transcription factors or are potentially involved in developmental processes (Table 3). A detailed analysis of these genes showed that only one *B. distachyon* gene (*Bradi4g40350*) has a wheat ortholog in the 5DS region completely linked to *VRN*-*D4.* This wheat gene, designated *TaAGL31* is a MADS-box gene similar to Arabidopsis homeotic floral gene *AGAMOUS.* Since other MADS-box genes such as *VRN1* play important roles in the regulation of flowering (Chen and Dubcovsky 2012), we initially hypothesized that *TaAGL31* might have played a similar role. However, no polymorphisms were detected in this gene between TDF-J and TDC carrying different *VRN*-*D4* alleles. In addition, no significant differences in *TaAGL31* transcript levels were detected between TDF-J and TDC in the leaves of 3- and 5-week-old plants (Fig. 4). Although we cannot rule out the existence of polymorphisms outside the sequenced region or differences in expression at time points that were not tested, the current results provide little support to the hypothesis that *TaAGL31* is a good candidate for *VRN*-*D4.*

Although we cannot rule out the possibility that another gene present in the *B. distachyon* chromosome region colinear with the wheat *VRN*-*D4* candidate region is the true homolog of *VRN*-*D4*, we favor the hypothesis that the *VRN*-*D4* gene originated in wheat after the divergence between wheat and *B. distachyon.* This hypothesis is supported by the lack of natural diversity for vernalization requirement in the wheat colinear centromeric regions on chromosomes 5AS and 5BS. In addition, the narrow geographical distribution of the dominant *Vrn*-*D4* (Iwaki et al. 2000, 2001) argues in favor of a recent origin of this gene/allele in hexaploid wheat.

### *VRN*-*D4* epistatic interactions

**Fig. 5:**
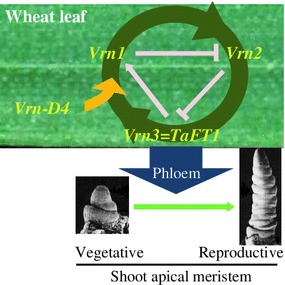
Hypothetical model of *VRN*-*D4* role in the wheat leaves. According to this model *VRN*-*D4* activates the *VRN1/VRN2/VRN3* positive feedback loop in the leaves, which results in an increase of *VRN3* (=*TaFT1*). The TaFT1 protein is then transported through the phloem to the shoot apical meristem where it induces the meristem-identity genes and the initiation of the reproductive stage

In a previous study we showed that in wheat, the presence of the dominant *Vrn*-*D4* allele in TDF-J was associated with a dramatic upregulation of *VRN1* and *VRN3* (=*TaFT1*) and downregulation of *VRN2* transcript levels in the leaves (Yoshida et al. 2010). The activation of the positive feedback loop formed by these three genes (Fig. 5) results in the accumulation of *VRN3* transcripts to very high levels (>tenfold *ACTIN*). The encoded TaFT1 protein is then transported to the shoot apical meristem where it induces *VRN1* [and likely its paralogs *FUL2* and *FUL3*, Chen and Dubcovsky (2012)] and initiates the transition from vegetative to reproductive stages.

Based on the previous expression results, we hypothesized that *VRN*-*D4* should be active in the leaves and should operate upstream (or be part) of the *VRN1/VRN2/VRN3* positive feedback loop (Fig. 5). The epistatic interactions observed in this study support this model (Fig. 3). In the presence of dominant *Vrn1* alleles that eliminate or greatly reduce the need of vernalization to activate this positive feedback loop, the addition of *Vrn*-*D4* has little additional effect on flowering time since its function in the leaves is redundant with that of *VRN1*. Similarly, when the dominant *Vrn*-*B3* is present, the high levels of *TaFT1* result in the upregulation of *VRN1* and the downregulation of *VRN2*, regardless of the presence of *Vrn*-*D4.* In wheat plants with a winter growth habit (*vrn1*, *Vrn2*, and *vrn3* allele combination) the positive feedback loop is not active. In this genetic background, the presence of the partially dominant *Vrn*-*D4* is sufficient to activate the loop, resulting in significantly earlier heading time than in the winter lines (Fig. 3). Since changes in the transcription profiles of any of the genes in this feedback loop result in the alteration of the transcription profiles of the other two, we currently do not know which of these genes is the potential target of *VRN*-*D4.*

To answer the previous question and to understand the molecular mechanisms involved in the *VRN*-*D4* controlled reduction of the vernalization requirement, we are currently attempting to clone the *VRN*-*D4* gene. In addition to the answers to these basic questions, the cloning of *VRN*-*D4* may have practical implications. *VRN*-*D4* is an important contributor to natural variation in flowering time and adaptation to several regions in Asia and therefore, the identification of the gene responsible for these differences may provide a useful tool to develop better adapted wheat varieties for a changing environment.

## Electronic supplementary material

Below is the link to the electronic supplementary material.

Supplementary material 1 (DOCX 28 kb)

## Abbreviations

RT-PCR: Reverse Transcription Polymerase Chain Reaction
QTL: Quantitative Trait Loci
