# Supplementary material for: Fine mapping and epistatic interactions of the vernalization gene VRN-D4 in hexaploid wheat
Source: Mol Genet Genomics. 2013 Nov 9;289(1):47–62. doi: 10.1007/s00438-013-0788-y (PMC3916708; doi:10.1007/s00438-013-0788-y)
Supplement: Supplementary file 1 — Supplementary material 1 (DOCX 28 kb) [file 438_2013_788_MOESM1_ESM.docx]

**Table S1.** Markers used to construct the *VRN-D4* physical map.

| **Wheat EST** | **Primer sequence (5' to 3')** | **Product size (bp)** | **Annealing temp. °C** | **Enzyme / indel** |
| --- | --- | --- | --- | --- |
| **BE591275** | F- CCTGAGAGTGAACCAGAGACT | 1098 | 62 | *Sac*I |
|  | R- ATACGCCTGGACTCCTCAGA |  |  |  |
| **BQ167501** | F- ccatcctcttaattgcgatac | 808 | 60 | Indel 3bp |
|  | R- catcgaacagataaacgtaag |  |  |  |
| **BE499257** | F- GGTTTGCGTTTGTGGAGTTT | 992 | 57 | *Hha*I |
|  | R- GCTACAGCATAAAAAGTTCACAG |  |  |  |
| **BE445181** | F- TATAGTGATTAGCAAATGATGGC | 683 | 57 | *Hae*III |
|  | R- CTTCCTTACATCCACGGCAT |  |  |  |
| **BE404490** | F- GTTTCCCACAAAGACGAGTAC | 876 | 56 | Sequence |
|  | R- CAACCCTTCTACTAACAGTA |  |  |  |
| **BE606654** | F- ACCGACAAGAACTCCTAGAT | 534 | 58 | *Bsa*AI |
|  | R- CAAAAGCATCGCAGAGAAACAC |  |  |  |
| **BG263382** | F- ACCCATGTGTACAAAATCTTCC | 1921 | 64* | *Bsp*HI |
|  | R- TCCAATCTCGTCCACAATGA |  |  |  |
| **TaAGL31** | F- AAGCTGCGCCACCAGATCCA | 704 | 64* | *Nsi*I |
|  | R- GGAACCGTCGAAGTTAATTTAATAGCCT |  |  |  |
| **BE403761** | F- CCGTCTGAATTATTTCACAGTC | 517 | 56 | Sequence |
|  | R- CAGTGCCCGTGCCAATCTC |  |  |  |
| **CJ717651** | F- CGGATAGTTCATACTTTTACTTG | 1736 | 64* | Sequence |
|  | R- CCATCATCGATAATGCTCAT |  |  |  |
| **CJ930261** | F- AGCGTTAGCCATCATCAAGC | 1839 | 66* | Sequence |
|  | R- AGGCAAGGCAGAAAGGAAAT |  |  |  |
| **CJ680715** | F- ATCTAGAATTACTATGGTAGGCAG | 1540 | 61* | Sequence |
|  | R- GTAAGTTCTTTACCAAGGTCAA |  |  |  |
| **CJ522104** | F- CTCTTGTAAACTTGCCAGCC | 1960 | 67* | Sequence |
|  | R- TCACCAGATTACACCGTCCA |  |  |  |
| **BJ315664** | F- ACTTGCGTTCCATGTTCACACTTGTAGG | 687 | 62* | *Taq*I |
|  | R- GCGCTCCTGGCAGCAGTCTC |  |  |  |
| **CJ521028** | F- TGGTTGTTGTACCTGCCTGAAAGTTAAGC | 786 | 59* | *Taq*I |
|  | R- CAGAAGGGAATTGTATTGTTTAAATGCAAATGGC |  |  |  |
| **TaFT-like** | F- CTCCATTGGTACGTGCTAAG | 1207 | 66* | *Bgl*I |
|  | R- GCGACTACACCCGCTTCTAC |  |  |  |
| **BE44702** | F- ACGATGTAAATGACTAGAATCTGTCTGAT | 210 | 60 | *Mn*I |
|  | R- GCTTCATCACCAAGGTCATGTGG |  |  |  |
| **BE444353** | F- GCCGTTTGATGTTGGCACGA | 922 | 63 | *Ssp*I |
|  | R- AGGGAATGTGACTGGAGGTG |  |  |  |
| **BE405060** | F- TGCACTTTCAGAATCGAAGCG | 454 | 60 | *Hha*I |
|  | R- GGGCTTCGCGTCTTCGTGA |  |  |  |

*94⁰C for 5 m, 94⁰C for 30 s, 8 cycles of initial TD (-0.5⁰C per cycle), followed by 40 cycles of normal PCR, final extension time of 7 min at 72⁰C. Marker BG313707 was previously published (Yoshida et al. (2010) and is not included in the Table.

**Table S2.** Primers and conditions for *VIL1* sequencing in diploid and hexaploid wheat.

| Amplified region | Primer | Sequence (5' to 3') | Product size (bp) ^a^ | Annealing  temperature (°C) | Annealing  time (sec) | Extension  time (sec) | Cycles |
| --- | --- | --- | --- | --- | --- | --- | --- |
|  |  |  |  |  |  |  |  |
| **Diploid wheat** |  |  |  |  |  |  |  |
| 5'UTR to intron1 | TmVIL1/5UTRF1 | GAGGCCAATTATGGCTGCTA | 1538 | 60 | 60 | 90 | 35 |
|  | TmVIL1/Ex2R | AACGTGGCAGGACGACTC |  |  |  |  |  |
| Exon1to exon2 | TmVIL1/WF2 | ATGGAGTCGACCGGAGGA | 1130 | 60 | 30 | 90 | 35 |
|  | TmVIL1/Ex2R | AACGTGGCAGGACGACTC |  |  |  |  |  |
| Exon2 to exon3 | TmVIL1/F2 | CTCATCGGAGACTGGTGATA | 719 | 57 | 60 | 90 | 38 |
|  | TmVIL1/Ex3R | AGGGGACCAACCTCAGTTTC |  |  |  |  |  |
| Exon3 to exon4 | TmVIL1/Ex3F1 | ATAGGCTTTTGGATGGCACA | 949 | 62 | 30 | 60 | 35 |
|  | TmVIL1/R3 | TCTCATTTCGGAGGCACTCT |  |  |  |  |  |
| Exon4 to 3'UTR | TmVIL1/F1 | AGGTTGCTCGTCTACTGCCA | 781 | 62 | 30 | 60 | 35 |
|  | TmVIL1/WR2 | AGTGTTTGCCTATCCGGATT |  |  |  |  |  |
| 3'UTR | TmVIL1/IPF1 | GTCTTGACCTTTATCCGCAC | 1367 | 60 | 60 | 90 | 35 |
|  | TmVIL1/3UTRR1 | GGCAGGTGGATTATTTGGTG |  |  |  |  |  |
| **Hexaploid wheat** |  |  |  |  |  |  |  |
| 5'UTR to intron1 | VIL1-up1060-F1 | CCCCCTCTAGACGCCTACCTTG | 1315（1314） | 65 | 60 | 90 | 35 |
|  | VIL-D1-int1-R3 | CACGCAGCATGGGACCCACCCA |  |  |  |  |  |
| Exon1 to intron1 | VIL-1-ex1-F1 | AGCGGAACCATCCGCTCTC | 264 (264) | 62.5 | 30 | 60 | 36 |
|  | VIL-D1-int1-R2b | GCGCGGGAGAAGAATGACCT |  |  |  |  |  |
| Intron1to exon2 | VIL-D1-int1-F1 | CAGCTTGCTTATTAGGCTACCATT | 656 (656) | 57 | 60 | 60 | 33 |
|  | VIL-D1-ex2-R2 | ACTGGCATCGTTTGATTCTAGC |  |  |  |  |  |
| Intron1 to intron2 | VIL-D1-T-int1-F1 | GCATATTCAAGTGTTCAACCAGAGT | 1001 (989) | 55 | 30 | 60 | 30* |
|  | VIL-D1-T-int2-R1 | CAACAAAACCTATCACAGAAAAGTACC |  |  |  |  |  |
| Exon2 to intron2 | TmVIL1/Ex2F1 | CTTGGGCAATCTATGCACCT | 537 (527) | 57 | 60 | 60 | 34 |
|  | VIL-D1-int2-R2 | AAAGGTACCATAAATAAGCAAAGCA |  |  |  |  |  |
| Intron2 to exon4 | VIL-D1-T-int3-F1 | TCTGTAAGAGCGTTTAGATCACCA | 823 (823) | 60 | 30 | 60 | 35 |
|  | TmVIL1/R2 | GATCCGAAAGGCATACTCCG |  |  |  |  |  |
| Entron3 to 3'UTR | VIL-D1-int3-F1 | GTGGGTTCATGTGGATTCTTTT | 1317 (1317) | 60 | 30 | 60 | 38 |
|  | VIL-D1-T-3UTR-R1 | CGAAGTGGAAAAACTGTGATACCT |  |  |  |  |  |
| Exon4 to 3'UTR | TmVIL1/IPF1 | GTCTTGACCTTTATCCGCAC | 768 (767) | 60 | 30 | 60 | 35 |
|  | VIL-D1-3UTR-R1 | CCAGAATTTAGCGGTGAGAAGA |  |  |  |  |  |
| 3'UTR | VIL-D1-3UTR-F1 | CACACAACAAAATGAAATGAGTGA | 826 (828) | 60 | 30 | 60 | 35 |
|  | TmVIL1/3UTRR1 | GGCAGGTGGATTATTTGGTG |  |  |  |  |  |

a: PCR product sizes were counted by using following sequences: For diploid wheat, *Triticum monococcum* sequence (DQ886922; Fu et al., 2007). For hexaploid wheat,

TDF-J (AB846583) and Hayakomugi (AB846584), and CS5402 (AB846585) in parenthesis. *94⁰C for 5 m, 94⁰C for 30 s, 10 cycles of initial TD (-0.5⁰C per cycle), followed

by 30 cycles of normal PCR, final extension time of 10 min at 72⁰C.
